# Comparing the clinical features of lateral and medial approaches of costoclavicular technique versus traditional lateral sagittal technique as infraclavicular brachial plexus block methods: a randomized controlled trial

**DOI:** 10.1186/s12871-024-02645-z

**Published:** 2024-07-25

**Authors:** Emre Sertaç Bingül, Mert Canbaz, Mehmet Güzel, Emine Aysu Şalvız, Bora Edim Akalın, Ömer Berköz, Ebru Emre Demirel, Zerrin Sungur, Meltem Savran Karadeniz

**Affiliations:** 1 Department of Anesthesiology and Intensive Care, Istanbul Faculty of Medicine, Istanbul University, Istanbul, Turkey; 2 Department of Anesthesiology, Regional Anesthesia & Acute Pain, Washington University in St Louis, School of Medicine, St. Louis, USA; 3 Department of Aesthetic, Plastic and Reconstructive Surgery, Istanbul Faculty of Medicine, Istanbul University, Istanbul, Turkey; 4 Department of Aesthetic, Plastic and Reconstructive Surgery, Division of Hand Surgery, Istanbul Faculty of Medicine, Istanbul University, Istanbul, Turkey

**Keywords:** Nerve blockade, Brachial plexus blockade, Upper extremity, Postoperative pain, Regional anesthesia, Acute pain

## Abstract

**Background:**

It is aimed to compare the block onset times and performance features of costoclavicular techniques (medial and lateral approach) versus lateral sagittal technique.

**Methods:**

Patients were randomized into three groups. For costoclavicular techniques, ultrasound probe was placed parallel to clavicle obtaining nerve cords, axillary artery and axillary vein visual from lateral-to-medial, respectively. The block needle was advanced from lateral (Group CLB) or medial (Group CMB) to perform costoclavicular block. For lateral sagittal technique (Group LSB), ultrasound probe was placed sagittal and perpendicular below the coracoid process to obtain sagittal artery image with the cords around. Total 20 ml of 0.5% bupivacaine and 10 ml of 2% lidocaine were deposited for all groups. Sensory and motor block onset times, block performance properties, complications, and patient/surgeon satisfactions were investigated.

**Results:**

Among 56 patients, the primary outcome, sensory block onset time was shorter in Group CLB than Group CMB and Group LSB (10 [5–15], 10 [10–20], and 15 [10–15] minutes, respectively, *p* < 0.05). Motor block onset was also fastest in Group CLB (15 [10–20] mins for CLB, 20 [15–20] mins for LSB, and 22.5 [15–25] mins for CMB, *p* = 0.004). Block performance properties did not differ between the groups. The only complication observed was vascular puncture with an incidence of 28% in Group CMB.

**Conclusions:**

Lateral approach costoclavicular technique provides fastest block onset than the other techniques. Considering the success and safety profile, this technique stands as a good alternative in clinical practice.

**Trial registration:**

This study is prospectively registered to clinicaltrials.gov on 20/02/2022 (NCT05260736).

## Introduction

With an average of 88% of success, infraclavicular brachial plexus block (ICB) is one of the many techniques that covers different surgical indications to anesthetize the upper extremity [1]. Approaching the brachial plexus from above or underneath the clavicle has always been a debate, and recent literature demonstrates a reduced risk of Horner’s syndrome with ICB despite being similarly safe and effective [1–3]. However, popularity of this block also originates from significantly less ulnar nerve sparing when performed in comparison to supraclavicular approach. Yet, the trials emphasizes that this phenomenon is valid when two or three injections are provided for ICB which means more needle maneuvers are required around the axillary artery in the lateral sagittal section of the plexus [2, 4].

In 2015, Karmakar et al. have suggested blocking the brachial plexus in the costoclavicular fossa as a “more suited” anatomy for ICB. Clustered anatomy of the cords in the fossa have been claimed as an upside which would result in faster onset of the block and “less” lower trunk sparing since “single” injection would be adequate [5]. Initial description of the costoclavicular brachial plexus block is from the lateral aspect (Costoclavicular lateral block: CLB), yet medial approach has also been defined (Costoclavicular medial block: CMB). Nieuwveld et al. has concluded that “medial” approach to costoclavicular block is anatomically feasible and effective even with lower volumes which raises the question for the best aspect to approach the costoclavicular space [6]. Some authors have reported manipulation difficulty with the needle in lateral costoclavicular approach due to the presence of coracoid process, and it might not be observed when approached medially [7]. Definitive studies regarding the clinical properties and performance features of these costoclavicular techniques are still sparse for such clinical assumptions, and lateral sagittal technique stands as a milestone to compare since it is preferred by many physicians and performed worldwide. The current study was exclusively planned to observe clinical features of costoclavicular approaches (medial and lateral separately) via comparing against traditional lateral sagittal approach.

## Materials and methods

In this clinical trial, it is aimed to compare the common clinical data such as block onset times and performance features of three abovementioned approaches of ICB which are lateral approach costoclavicular block (CLB), medial approach costoclavicular block (CMB), and also the lateral sagittal infraclavicular approach (LSB). The hypothesis is that the sensory block onset time would be shorter with lateral approach costoclavicular technique in comparison to lateral sagittal technique. Therefore, the primary outcome was “sensory block onset time”. Secondary outcomes included motor block onset time, ideal ultrasound (US) visualization time, duration of block procedure, perceived difficulty of needle tip and shaft visualization, perceived difficulty of total block performance, number of needle maneuvers, need for additional needle maneuver, total number of local anesthetic (LA) injections, complications (e. g. pneumothorax, vascular punctures, diaphragm paralysis), postoperative pain scores and length of stay.

### Study population and regulatory aspects

Following the approval of institutional ethical approval (Istanbul University Istanbul Faculty of Medicine Clinical Research Ethics Committee-2022/151) and registry to clinicaltrials.gov (www.clinicaltrials.gov, NCT05260736, 20/02/2022), adult patients who were scheduled for upper extremity surgery in our Hand Surgery Division of Aesthetic, Plastic and Reconstructive Surgery Department and planned to undergo infraclavicular brachial plexus block anesthesia were enrolled in the investigation. Written informed consent was provided from every patient before the enrollment. Accordingly, patients older than 18 years of age with an American Society of Anesthesiologists Physical Status (ASA) between I to III who were planned to have arm, forearm, and hand surgery were included. Exclusion criteria were existing bleeding diathesis, or being under anticoagulant therapy, or existing local infection on the intervention area, or known LA allergy.

### Study design

This study was planned as a single center, randomized, clinical trial. The randomization was generated via numbered, sealed, opaque envelopes in which the designated groups (Group CLB, Group CMB, and Group LSB) were written. The envelopes were prepared according to web-based randomization (www.sealedenvelope.com, seed no:84069950826186) that was prepared by an independent researcher prior to the patient enrollment to the study. In the morning of the operation, the envelopes were opened by the operating anesthetists (EAB, MSK, EED) whose regional anesthesia experience is over 5-years, and the designated brachial plexus block were implemented accordingly. Block performance data were recorded by another researcher who was not blinded to the procedure. The perioperative clinical follow-ups (motor and sensory examinations, pain scores) were provided by blinded researchers.

### Interventions

After arrival to the operating room, an intravenous line was inserted onto the nonsurgical arm, and standard monitoring with electrocardiogram, pulse oximetry, and noninvasive blood pressure was applied. All the patients were premedicated with midazolam 0.05 mg/kg IV and fentanyl 1 mcg/kg IV prior to the procedure, and the arms were abducted 90° to the body before the initiation of all blocks.

**Fig. 1:**
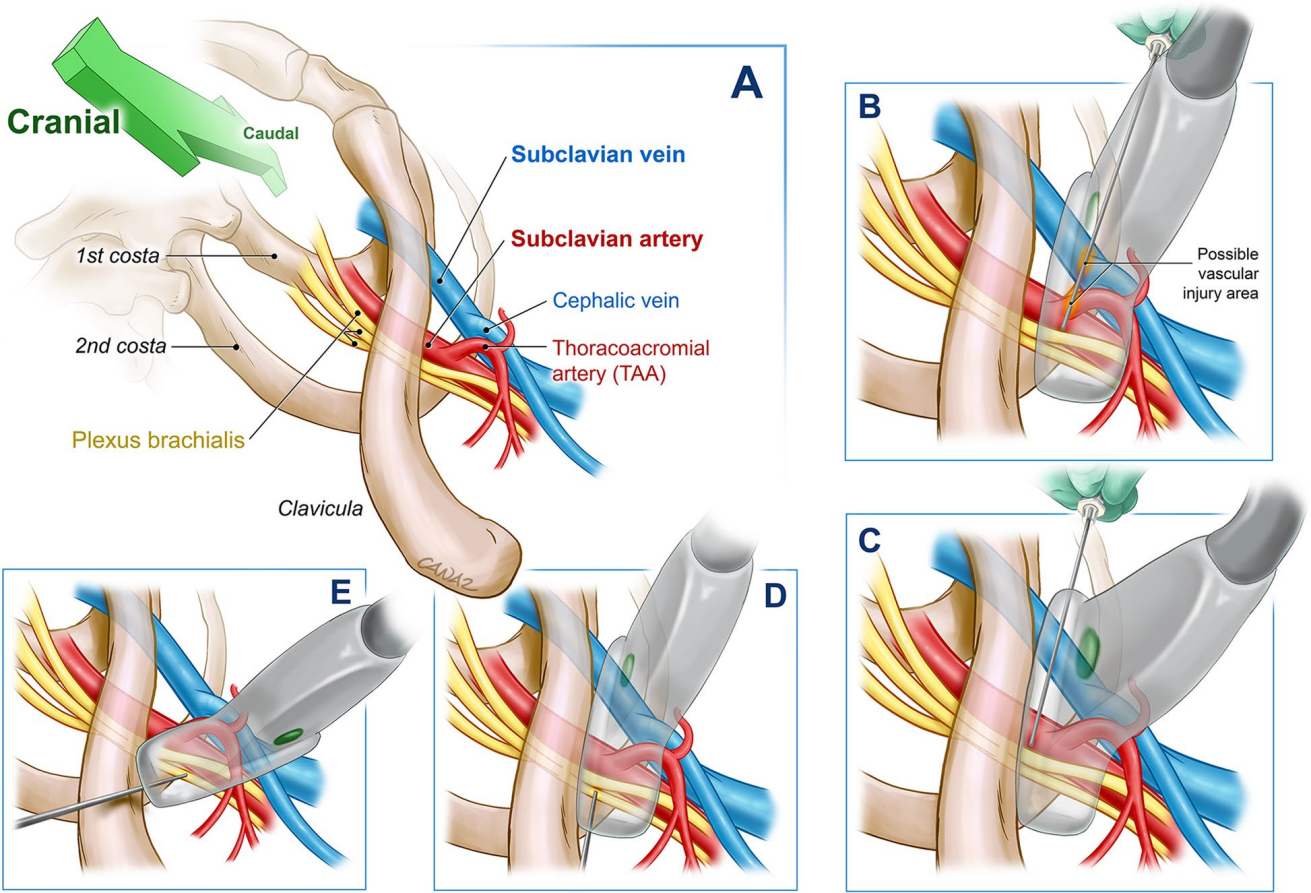
Explanatory illustration of the three different infraclavicular brachial plexus block techniques. **A** Anatomy of the block region, **B** Vascular tissues that are close to needle trajectory in medial approach costoclavicular technique, **C** Ideal imaging and correct technique for costoclavicular medial block (The ultrasound probe is tilted to more cranially), **D** The technique for costoclavicular lateral block, **E** The technique for lateral sagittal infraclavicular block

**Fig. 2:**
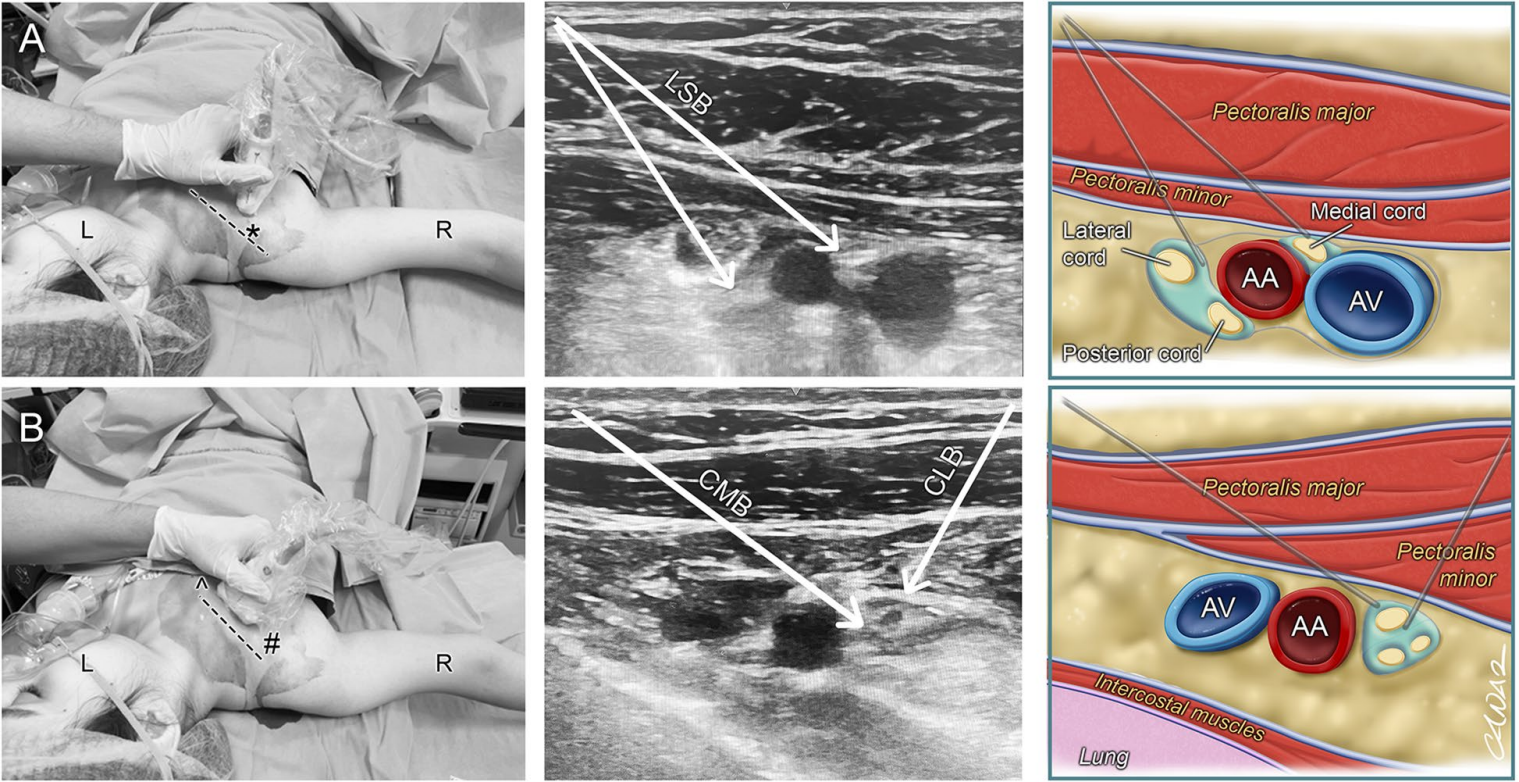
Brief demonstration of three different block techniques. L: Left, R: Right. **A** Lateral sagittal infraclavicular brachial plexus block. LSB: Lateral sagittal approach infraclavicular block needle direction, AA: Axillary Artery, AV: Axillary Vein, *: Puncture site for the technique. **B** Lateral and medial approaches of the costoclavicular brachial plexus block. CMB: Medial approach costoclavicular brachial plexus block needle direction, CLB: Lateral approach costoclavicular brachial plexus block needle direction. ^: Medial approach costoclavicular block puncture site, #: Lateral approach costoclavicular block puncture site

For LSB, a linear US probe (5–13 MHz, GE Healthcare, USA) was placed sagittal and perpendicular to the skin on the distal part of clavicle, aligned with the coracoid process [8]. Ideal visualization included pectoralis major muscle, pectoralis minor muscle, axillary artery pulsating from superior to inferior, and axillary vein aside. Total 20 cc of bupivacaine 0.5% and 10 cc of lidocaine 2% were made as divided injections via a 100 mm insulated peripheral block needle (Sonoplex II, 22 Ga, Pajunk, Germany) which was advanced targeting 7 and 1 o’clock of the artery for LA deposit. For CLB, US probe was placed parallel to the clavicula on the midpoint and tilted cranially which provided the costoclavicular fossa image. Ideal image was considered as axillary vein, axillary artery, and the brachial plexus cords (lateral, medial, and posterior cord) from medial to lateral. Block needle was advanced from the lateral aiming the center of the cluster where a single injection was intended to deposit 20 cc bupivacaine 0.5% and 10 cc of lidocaine 2% [5]. For CMB, under the guidance of same ultrasonographic view, again aiming for the center of the neural cords, the needle was advanced from medial-to-lateral passing by axillary vein on the most medial, and axillary artery in the middle [6]. The same LA doses were valid for this block also. Ideal ultrasonographic view for CMB was accepted as the one which is without the presence of thoracoacromial artery and cephalic vein that are in connection with axillary artery and axillary vein, respectively. Extra LA injections were provided in case the spread was considered not adequate. Of note, injection safety was provided via using pressure limiting valves (NerveGuard, Pajunk, Germany) attached to the block needle to prevent neuropraxis. Vascular puncture was avoided via applying negative aspiration prior to every 4 ml of LA injection, and if blood was encountered in the syringe, the needle was drawn back until blood is not aspirated and redirected to where a safe injection would be provided. All the patients were followed-up considering possible toxicity findings such as agitation, lethargy, dysrhythmias, hemodynamic instabilities. The abovementioned block techniques are summarized in Figs. 1 and 2. Postoperative analgesia protocol consisted of paracetamol 1 g IV twice a day starting of at 20:00 on the surgery day as a routine practice, and if the NRS was above “3” thirty minutes after paracetamol administration, additional tramadol 0.5 mg/kg IV was planned to be administered.

### Outcome measures

Duration of ideal US view visualization and the procedure (seconds), perceived difficulty of needle visualization, and total procedure (5-point Likert Scale; 1: very difficult, 2: difficult, 3: average, 4: easy, 5: very easy), total number of needle maneuvers and injections, number of vascular punction, and sensory and motor block onset times (minutes) were recorded throughout the intervention process.

The primary outcome “sensory block onset time” and secondary outcome “motor block onset time” were examined on 0th, 5th, 10th, 15th, 20th, 25th, 30th, and 45th minute via checking the brachial plexus terminal nerves, separately: *median nerve, ulnar nerve, radial nerve,* and *musculocutaneus nerve.* For the sensory evaluation, a blunt needle was pinned on the relevant dermatomal areas of the branches (*median nerve*: volar face of the middle finger, *ulnar nerve*: volar face of the 5th finger, *radial nerve*: hand dorsum and *musculocutaneus nerve*: lateral part of forearm), and scored as follows: 0: feels pain/absent sensorial blockade, 1: feels touch/partial blockade, 2: no sense/complete blockade. Motor activity was evaluated via checking the motor response of the same branches (*median nerve*: hand flexion or thumb opposition, *radial nerve*: hand dorsiflexion or thumb abduction, *musculocutaneus nerve*: elbow flexion, *ulnar nerve* thumb adduction), and accordingly scoring was as follows: 0: no motor block, 1: partial motor block, 2: total motor block. For the both physical examinations, a score of > 6/8 was considered adequate block which refers to “readiness for surgery”, and the time for reaching adequate sensory block was considered “sensory block onset time”.

Once the surgery was finished, numeric rating scales for postoperative pain (0–10), length of stay, and sensory and motor block recovery times (minutes) was obtained by blinded researchers. Patients and surgeons were asked to rate their satisfaction. Their response was evaluated as follows; 0: Not satisfied, 1: Little satisfied, 2: Satisfied 3: Very satisfied. Furthermore, any postoperative complications, such as pneumothorax, hemothorax, diaphragm paralysis, vascular puncture, and Horner’s syndrome were recorded. For detection of such complications, clinical follow-up and physical examination were chosen, and necessary further imaging and evaluation were provided upon clinical signs and symptoms.

Based on Songthamwat et al.’s study [9], and assuming a 45% shorter sensory block onset with CLB in comparison to LSB (10 ± 7 vs 18 ± 7 min, respectively), 18 patients per group was estimated with an alpha value of 0.05 and power of 90% using computer generated tool (G*Power 3.1, Dusseldorf, Germany). With the possibility of 10% drop-out, total 60 patients were planned to be enrolled in the investigation.

### Statistical analysis

Kolmogorov–Smirnov test used for the groups to exhibit the normality of the data distribution. Homogenous data were evaluated via Student’s t-test or ANOVA for binary or triple group comparisons, and heterogenous data were evaluated via Mann Whitney-U or Kruskal Wallis Test for binary or triple group comparisons, respectively. Categorical data were expressed using Chi-Square and Fischer’s exact test if needed. Mean ± standard deviation (SD) was used to define normally distributed data, and Skewed data were presented as median and interquartile range (IQR). The analyses were executed two-tailed, and *p* < 0.05 was considered significant. The SPSS for Mac version 21 (SPSS Inc, Chicago, Illinois) software package was used for statistical analyses.

## Results

**Fig. 3:**
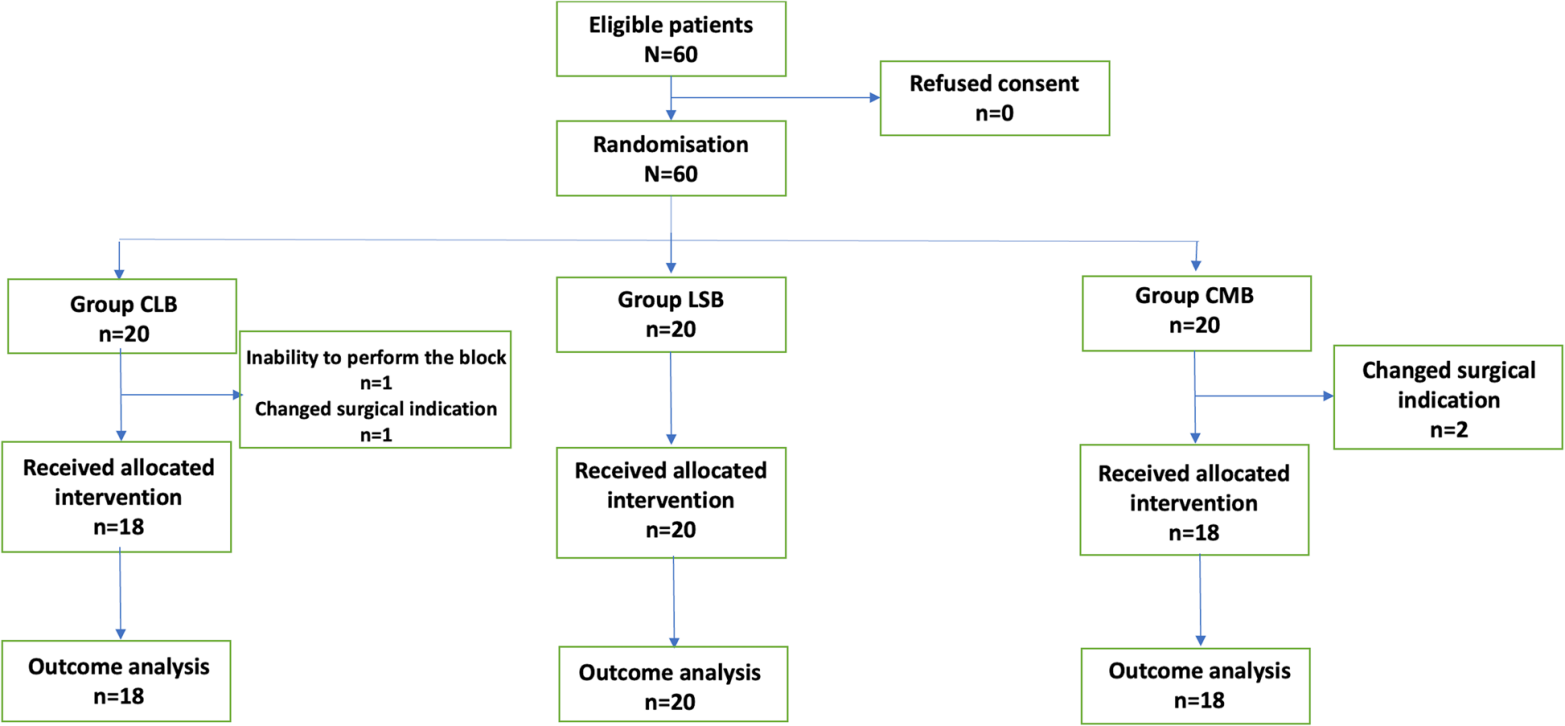
The CONSORT diagram. CLB: Costoclavicular Lateral Block, CMB: Costoclavicular Medial Block, LSB: Lateral Sagittal Block

Sixty patients were enrolled in the study between June 2022 and May 2023. Among these, 1 patient from Group CLB, and 2 patients from Group CMB were excluded due to changed surgical indication before starting to surgery. One patient from CLB group was also excluded due to anatomical impossibility of proceeding the needle into the aimed area which consequently required change of block technique. Therefore, the investigation was completed with 56 patients. The CONSORT diagram is presented in Fig. 3. Demographic data were similar in all groups except for the ex-smoker numbers (*p* = 0.01) (Table 1). Surgery types and durations did not differ between the groups (*p* > 0.05). The block success was 100% for all groups.

**Table 1:** Demographics and surgical features of the patients

	Total (***N*** = 56)	CLB (***n*** = 18)	CMB (***n*** = 18)	LSB (***n*** = 20)	***p*** value
Age (years)	39,9 ± 13,8	43,2 ± 14,1	38,0 ± 15,1	38,6 ± 12,6	0,5^a^
Gender (n, %)					
Female	30, 54%	8, 44%	11, 61%	11, 55%	0.6^b^
Male	26, 46%	10, 56%	5, 39%	9, 45%	
BMI (kg/m^2^)	27.8 ± 5.1	28.7 ± 3.6	27.5 ± 5.5	27.4 ± 5.9	0.7^a^
BMI > 30 (n, %)	17, 30%	7, 39%	5, 28%	5, 25%	0,6^b^
ASA					
I	29, 52%	7, 39%	7, 39%	15, 75%	0.07^b^
II	25, 45%	11, 61%	10, 56%	4, 20%	
III	2, 4%	0, 0%	1, 5%	1, 5%	
Comorbidities;					
*Ex-smoker*	16, 29%	7, 39%	8, 44%	1, 5%	0.01^b^
*Diabetes Mellitus*	8, 14%	5, 28%	2, 11%	1, 5%	0.1^b^
*Hypertension*	6, 11%	1, 6%	4, 22%	1, 5%	0.2^b^
*Coronary heart disease*	1, 2%	0, 0%	0, 0%	1, 5%	0.4^b^
Surgery (n, %);					
*Pulley finger*	3, 5%	1, 6%	0, 0%	2, 10%	0.4^b^
*Carpal tunnel syndrome*	12, 22%	4, 22%	3, 17%	5, 25%	
*Mass excision*	18, 32%	8, 44%	4, 22%	6, 30%	
*Tendoplasty*	18, 32%	3, 17%	8, 44%	7, 35%	
*Contracture release* + *graft*	5, 9%	2, 11%	3, 17%	0, 0%	
Surgery duration (minutes)	52.5(40–85)	50(38.6–85)	52.5(43.8–96.3)	55.0(40–80)	0.6^c^

Data are presented as mean ± standard deviation or median(interquartile range)

*CLB* Costoclavicular lateral block, *CMB* Costoclavicular medial block, *LSB* Lateral sagittal block, *ASA-PS* American Society of Anesthesiologists Physical Status, *BMI* Body mass index

^a^ANOVA test

^b^Pearson Chi-Square test

^c^Kruskal Wallis test

**Fig. 4:**
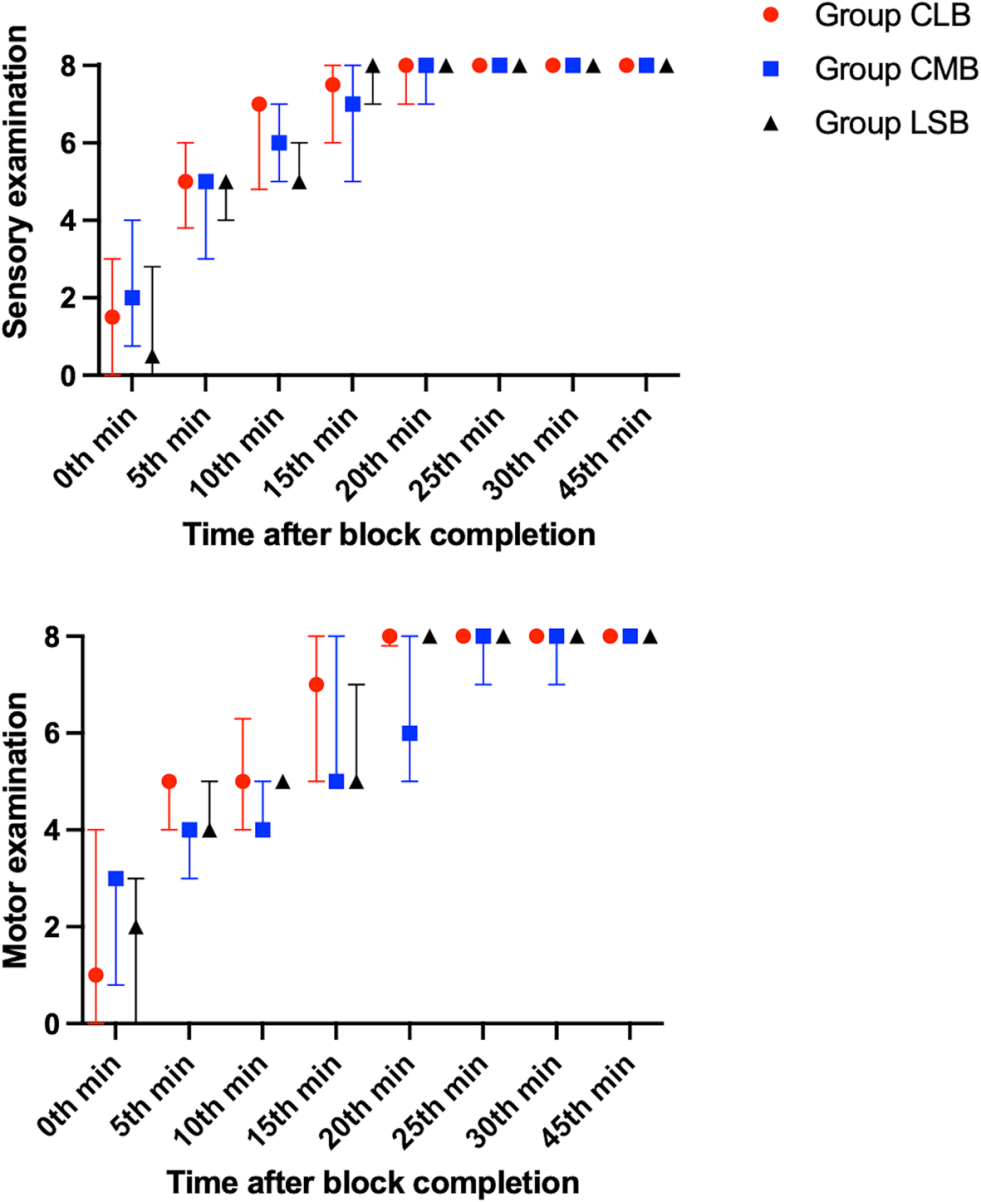
Sensory and motor block examinations for all groups after block completion. CLB: Costoclavicular lateral block, CMB: Costoclavicular medial block, LSB: Lateral sagittal block

Sensory block onset was fastest with lateral costoclavicular approach (*p* = 0.01). Post-Hoc analyses demonstrated significantly shorter block onsets with CLB when separately compared to CMB and LSB (10 [5-15], 10 [10–20], and 15 [10–15] minutes, respectively, *p* < 0.05). Furthermore, CLB represented the fastest motor block onset times which were again statistically significant in binary comparisons with Post-Hoc analyses (15 [10–20] mins for CLB, 20 [15–20] mins for LSB, and 22.5 [15–25] mins for CMB, *p* = 0.004). Block onset trends for groups were exhibited in Fig. 4. Ideal US visualization times and total procedure times were not different between the groups, and Likert scores were statistically close in all groups for needle tip and shaft visualization, and also total procedural difficulty (*p* > 0.05). Moreover, total number of needle maneuvers, need for additional needle maneuver, and total number of injections were statistically similar (*p* > 0.05). The only complication regarding the block technique was vascular puncture throughout the investigation. In 7 cases, vessels were punctured unintentionally and 5 of them were in Group CMB. Length of stay at hospital data were also similar for all groups, and postoperative nausea-vomiting incidences were close (*p* > 0.05). All the above-mentioned data are summarized in Table 2.

**Table 2:** Block performing features and comfort parameters

	CLB (*n* = 18)	CMB (*n* = 18)	LSB (*n*= 20)	*p* value
Sensory block onset time (minutes)	10(5–15)^c^	10(10–20)	15(10–15)	**0.01**^**a**^
Motor block onset time (minutes)	15(10–20)^c^	22.5(15–25)	20(15–20)	**0.004**^**a**^
Ideal USG visual obtaining time (seconds)	20.5(15–28.6)	24(12.5–27.5)	16.5(10–21.5)	0.2^a^
Time spent for block performance (seconds)	237.5(176.3–288.8)	204.5(175–246.3)	297.5(185–347.5)	0.2^a^
Needle tip visualization difficulty (Likert scale)	4(2–4.3)	2(2–4)	4(3–4)	0.06^a^
Needle shaft visualization difficulty (Likert scale)	3.5(2–4.3)	2(2–4)	4(3–4)	0.1^a^
Procedural difficulty perceived by the operating anesthetist (Likert Scale)	3.5(3–4.3)	2.5, (2–4)	3.5(2.3–4)	0.1^a^
Number of needle maneuvers (n)	2(2–3)	2.5(2–4)	3(3–4)	0.07^a^
Need for additional needle maneuver due to insufficient LA spread (n, %)	15, 83%	10, 56%	14, 70%	0.2^a^
Total number of LA injections (n)	2(1.8–3)	2(2–3)	3(2–3)	0.1^a^
Vascular puncture (n, %)	1, 6%	5, 28%	1, 5%	
Postoperative nausea vomiting (n, %)	1, 6%	1, 6%	0, 0%	0.6^a^
Length of stay (hours)	20.5(19.8–24)	24(21–25)	23.5(21.3–25)	0.07^a^
Patient Satisfaction score	3(3–3)	3(2–3)	3(2–3)	0.7^a^
Surgeon Satisfaction score	3(3–3)	3(2–3)	3(3–3)	0.9^a^

Data are presented median(interquartile range). Satisfaction score: 0 = Not satisfied, 1 = Little satisfied, 2 = Satisfied 3 = Very satisfied

Likert scale indicates range between 1 to 5 (1: Very difficult, 5: Very easy)

*CLB* Costoclavicular lateral block, *CMB* Costoclavicular medial block, *LSB* Lateral sagittal block, *USG* Ultrasonography

^a^Kruskal Wallis test

^b^Pearson Chi-Square test

^c^Significant difference was detected between CLB versus LSB, and CLB versus CMB groups in post-hoc analyses

**Fig. 5:**
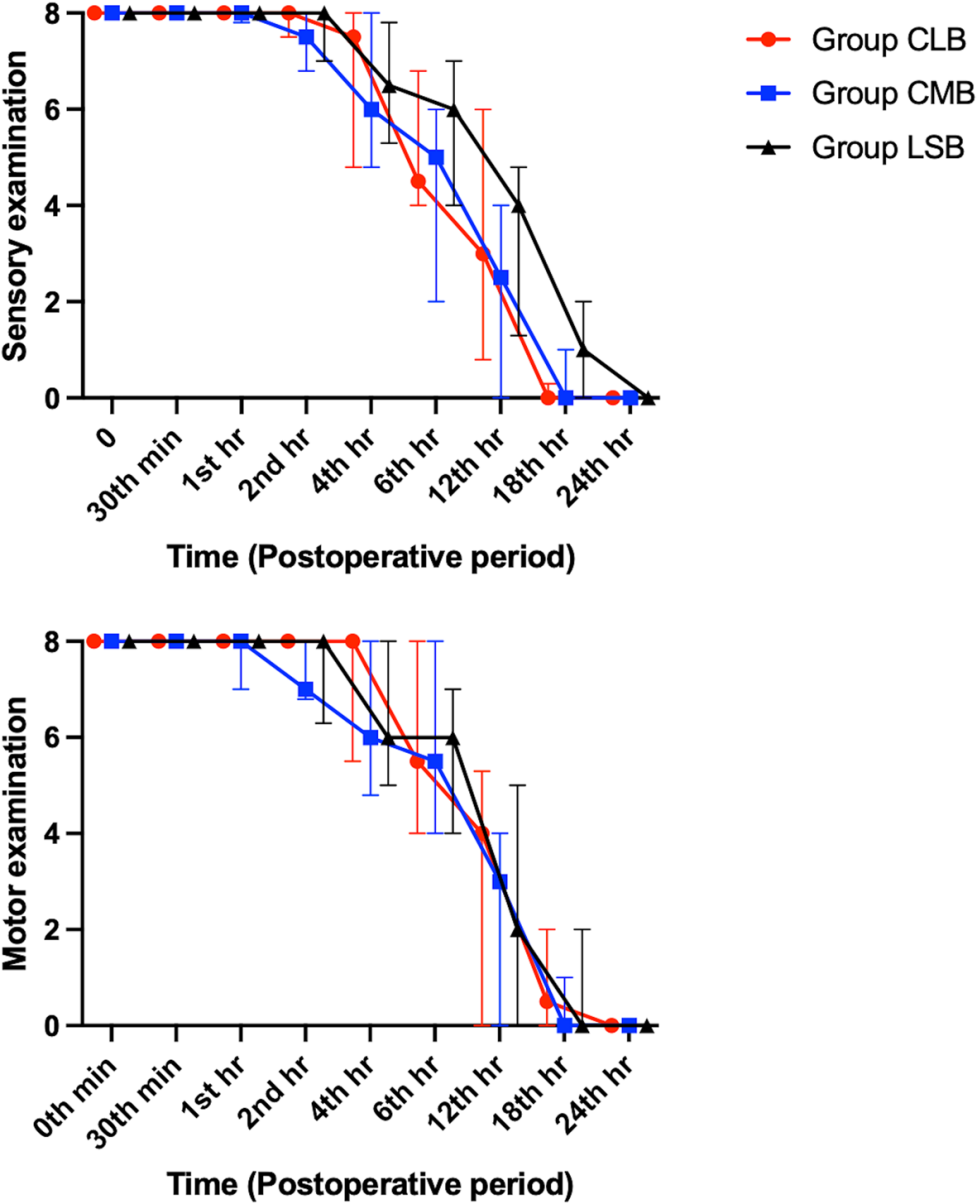
Postoperative sensory and motor block examinations for all groups. CLB: Costoclavicular lateral block, CMB: Costoclavicular medial block, LSB: Lateral sagittal block

Postoperative sensory and motor examinations did not represent any difference at any time points which is summarized in Fig. 5. NRS scores were similar for all the groups (Table 3). Of note, tramadol was not needed in the postoperative analgesia management in any of the groups.

**Table 3:** Postoperative Numeric Rating Scales for pain (NRS)

NRS	Group CLB	Group CMB	Group LSB	p
0th min	0(0–0)	0(0–0)	0(0–0)	0,3^a^
30th min	0(0–0)	0(0–0)	0(0–0)	0,3^a^
1st hour	0(0–0)	0(0–0)	0(0–0)	0,4^a^
2nd hour	0(0–0)	0(0–0)	0(0–0)	0,4^a^
12th hour	2(1.8–4)	1.5(0–3)	2.5(2–3)	0,2^a^
24th hour	2(1–2)	1(0.5–1.5)	1.5(1–2)	0.06^a^

Data are presented as Median(Interquartile range)

^a^Kruskal Wallis Test

## Discussion

In this randomized clinical upper extremity block trial, which compared the newest techniques of costoclavicular block versus traditional lateral sagittal technique, significantly faster sensory and motor block were observed with lateral costoclavicular approach while representing similar technical difficulty and postoperative comfort with the other techniques. This faster block onset may be interpreted as an advantage of injection into a clustered area via a shorter lateral needle course which allows LA deposition in a more clinically practical way causing more rapid onset [10]. Another reason would be the increased nonneural tissue/neural tissue ratio within the epineurium on the lateral parts of brachial plexus when compared midinfraclavicular to subcoracoid regions [11].

Brachial plexus blocks are pragmatic and time-efficient anesthetic methods for upper extremity surgeries that may enhance operating rooms’ turn-over if the facilities are adequate, and reliability, a good safety profile and practicality are the most desired features for such blocks. As a recently developed technique, costoclavicular brachial plexus block has been gaining popularity because of the hypothetical anatomical advantages which still need testing in the clinical set-up [5, 12, 13]. Data regarding the performance properties of these block (lateral or medial approach costoclavicular block) are still sparse, and despite this sparsity, high quality dose-defining studies exist which bring an insight for performing the block, and the recommended volumes (e.g. 20 cc of ropivacaine 0.5%) were chosen for this clinical trial [14–16]. A median 10 min duration of sensory block onset data was in line with the majority of existing clinical trials’ results [9, 17–20]. Yet, one of the few studies comparing CLB versus LSB, Leurcharusmee et al*.* did not observe significant difference between the groups which is contrary to ours and Songthamwat et al*.*’s results [9, 20]. In the current study, “block onset” was taken as “readiness” to surgery by accepting 7 points out of 8 as “adequate” for operation. This may also alter the presented data as it is seen in Aliste et al.’s study in which the sensory onset time was described as 21.6 min mean, because the authors analyzed only patients with complete block at 30th minute of the intervention [21]. Around 20 min of “surgery readiness” time is favorable for clinical practice, and such rapid onsets represent high value for the clinics with high patient volume. Of note, even 5 min of block onset was described with CLB in the literature which is at least two-fold faster than those presented above [22, 23].

The results of the current study also revealed faster block onsets with CLB when compared to CMB, even though the median values are the same. This interesting result may arise from the fact that the block needle follows a steeper path in lateral approach which leads a better targeting for the center of neural cluster. On the other hand, needle trajectory is more flattened in medial technique which may occasionally disrupt aiming the center. This is compatible with the presented data since every type of block in the current study required additional injections to cover intended areas, and among them CLB would be the best approach to obtain most appropriate injection site and fastest block onset.

Having the neural cords clustered in the costoclavicular fossa may arise the idea that single LA injection would be adequate to provide surgical anesthesia. However, as seen in our results, almost 70% of the cases required additional injections because the LA did not spread to intended areas. In line with this, previous studies have had demonstrated the superiority of multiple injections over single injection method for CLB [24, 25]. This might be due to the existence of a septae between the lateral cord and the other cords which requires further anatomical investigations [24, 26, 27]. However, in a cadaver study by Sala-Blanch et al*.*, it is underlined that there is a dissection requiring firm apposition between the medial and the posterior cords which may be the cause lower success with single injection methods [28]. Therefore, the hypothetical advantage of CLB regarding the success with less injections compared to LSB seems to be invalid.

Another debate would be on choosing the most favorable side for punction to reach the costoclavicular space (CCS). Some clinical trials assumed that lateral approach may occasionally be complicated by the coracoid process which distorts the needle manipulation [7]. We have observed this incident in only one patient to whom CLB could not been done due to low body weight. The patient’s BMI was 19 and coracoid process was relatively protuberant blocking the physician’s needle maneuver which consequently led impossible LA deposit to the CCS. Since Nieuwveld et al*.* described the medial approach costoclavicular block, quite few data have been presented in the literature [6]. The current study also provides an insight regarding the clinical usage of CMB. As it is demonstrated here, in extremely thin patients whose coracoid process is upfront enough to complicate manipulation, medial approach can be chosen instead of lateral approach. Because of the similar block success and postoperative pain scores, CMB stands as a good alternative. However, anatomical concerns exist. The ultrasound visualization of the CCS requires further expertise if the needle is advanced from medial to lateral, because thoracoacromial artery, cephalic vein or aberrant vascular tissues like superior thoracic artery may take place on the needle trajectory as it is demonstrated in Fig. 1B in the current manuscript [29, 30]. Vascular punction was observed in 28% of our CMB group patients whereas the ratio was 6% in the other groups. We were not able to demonstrate a significant relation, because the sample size was not calculated according to this specific complication, initially. However, the tendency to more unintentional vessel puncture with CMB seems solid. Moreover, the poorer Likert scores in CMB group supports the idea, however we could not demonstrate the significance here, also. Of note, the comparison of the incidence of vascular puncture between CLB and LSB was reported clinically nonsignificant in the literature [9, 20]. Lateral approach costoclavicular block represents favorable complication profile. As we have demonstrated in our previous study, the risks of such complications like pneumothorax, diaphragm paralysis, and hemothorax are remarkably low, even in pediatric patients [31].

As a solid limitation regarding to this pragmatic clinical trial is being a single center study, more patients would help enlightening and comparing the safety profile of these blocks. Thereby, it would reflect the complication potential of costoclavicular techniques, especially the medial approach. On the other hand, the rest of the data are accepted as favorable under clinical terms.

With the apparent properties, lateral approach costoclavicular block may be considered as a strong choice among the infraclavicular techniques. Its rapid-acting and easy-to-perform features are not inferior to lateral sagittal block which is used widely in the anesthesiology practice. Since it is a different concept, recognizing costoclavicular space and the surrounding anatomical structures, and performing the block would require more expertise. Perhaps, performing such variable blocking techniques brings more adaptation to individual sonoanatomy.

## Data Availability

The clinical data of this trial is available upon reasonable request to the corresponding author.
